# No Consistent Effect of *ADRB2* Haplotypes on Obesity, Hypertension and Quantitative Traits of Body Fatness and Blood Pressure among 6,514 Adult Danes

**DOI:** 10.1371/journal.pone.0007206

**Published:** 2009-09-25

**Authors:** Anette P. Gjesing, Thomas Sparsø, Knut Borch-Johnsen, Torben Jørgensen, Oluf Pedersen, Torben Hansen, Niels V. Olsen

**Affiliations:** 1 Hagedorn Research Institute, Gentofte, Denmark; 2 Steno Diabetes Center, Gentofte, Denmark; 3 Institute of Biomedical Sciences, University of Copenhagen, Copenhagen, Denmark; 4 Research Centre for Prevention and Health, Glostrup University Hospital, Glostrup, Denmark; 5 Department of Neuroanaesthesia, Copenhagen University Hospital, Copenhagen, Denmark; 6 Faculty of Health Science, University of Aarhus, Aarhus, Denmark; 7 Faculty of Health Sciences, University of Southern Denmark, Odense, Denmark; Ohio State University Medical Center, United States of America

## Abstract

**Background:**

Evidence regarding the association of variation within *ADRB2*, the gene encoding the beta-adrenergic receptor 2 (ADRB2) with obesity and hypertension is exceedingly ambiguous. Despite negative reports, functional impacts of individual genetic variants have been reported. Also, functional haplotypes as well as haplotype combinations affecting expression levels *in vivo* of ADRB2 mRNA and protein as well as receptor sensitivity have been reported. The aim of the present study was therefore to evaluate if variations within *ADRB2* as haplotypes or as haplotype combinations confer an increased prevalence of obesity and hypertension among adults.

**Methodology/Principal Findings:**

We genotyped five variants required to capture common variation in a region including the *ADRB2* locus in a population-based study of 6,514 unrelated, middle-aged Danes. Phases of the genotypes were estimated *in silico*. The variations were then investigated for their combined association with obesity, hypertension and related quantitative traits. The present study did not find consistent evidence for an association of *ADRB2* variants with either obesity or hypertension when variations were analysed in a case-control study. The same lack of impact was also seen in the quantitative trait analyses, apart from nominal differences on waist-to-hip ratio and systolic blood pressure between specific haplotype combinations.

**Conclusions/Significance:**

In a population-based sample of 6,514 Danes we found no consistent associations between five common variants which tag the *ADRB2* locus and prevalence of obesity or hypertension neither when analysed as individual haplotypes nor as haplotype pairs.

## Introduction

The beta-adrenergic receptor 2 (ADRB2) is part of the sympathetic nervous system influencing metabolic as well cardiovascular functions. Due to this biological function, ADRB2 has been a candidate for the development of obesity and hypertension; however, no consistent associations with these conditions have been observed. Previous studies have especially examined two functional variants, the Arg16Glu and the Gln27Glu, which in few studies were associated with both obesity [1], [2] and hypertension [1], [2]. However, overall there is ambiguity regarding the effect of these variants and in our previous study none of them were associated with either trait [3].

In addition to functional single variations, functional haplotypes have also been reported within this locus. One study identified that two common haplotypes, composed of 13 single nucleotide polymorphisms (SNPs) located within the *ADRB2* locus, displayed a significantly different expression of both mRNA and protein in HEK cells [4]. Furthermore, the response to inhalation of the ADRB2 agonist albuterol was different depending on the combinations of these common haplotypes [4]. Haplotypes based on 10 of these SNPs have also showed significantly lower sensitivity as well as a lipolytic effect in response to noradrenalin stimulation [5]. In addition, a study identified that only four of the 13 SNPs included in the study by Drysdale and colleagues accounted for the majority of the haplotypic differences [6]. Also, haplotypes composed of these four SNPs were associated with obesity in a case-control study comprising 59 obese, 61 overweight and 79 lean individuals [6]. Moreover, *ADRB2* haplotypes were reported to have a physiological effect on measures of blood pressure [7]. Thus, despite the previous lack of a consistent effect of individual SNPs in *ADRB2*, there are several indications that haplotypes and haplotype combinations may have a physiologically significant effect, yet the effect of haplotypic pairs is so far unexplored in a large-scale study sample.

Therefore, we examined the impact of the individual haplotypes and haplotypic combinations within *ADRB2*. Five SNPs were selected using pair wise tagging with an R^2^ above 0.8 as they were required to represent variation within the *ADRB2* locus covering a 15 Kbp region which included *ADRB2* and two flanking linkage disequilibrium (LD) blocs according to HapMap (http://www.hapmap.org). This was followed by analyses of the effect of haplotypes and haplotype combinations in relation to the prevalence of obesity and hypertension.

## Materials and Methods

### Study sample

We studied a Danish population-based study sample involving of 6,514 middle-aged Danes from the greater Copenhagen area sampled at Research Centre for Prevention and Health (*n* = 6,514; 3,169 men, 3,345 women) aged 46±8 years (mean±SD) and having a (body mass index) BMI of 26.3±4.6 kg/m^2^ (ClinicalTrials.gov ID-no:NCT00289237 [8]). Hypertension was defined as mean systolic blood pressure≥140 mm Hg and/or mean diastolic blood pressure≥90 mm Hg and/or current or previous treatment with antihypertensive drugs. Obesity was defined as individuals having a BMI above 30 kg/m^2^. A lean control individual was defined as having a BMI less the 25 kg/m^2^.

### Ethics statements

Informed written consent was obtained from all subjects before participation. The study was approved by the Ethical Committee of Copenhagen County and was in accordance with the principles of the Helsinki Declaration.

### Anthropometrics and physiological measures

Height and weight were measured in light indoor clothes and without shoes, and BMI was calculated as weight (kg)/height (m)^2^. Waist circumference was measured at the umbilical level to the nearest 0.5 cm with the subjects standing using a non-extendable linen tape measure according to WHO recommendation. The remaining measures were performed as previously described [9].

### Genotyping

rs1042713, rs1042714, rs1800888, rs1042718 and rs1042719 were selected for analysis of the *ADRB2* locus using pair wise tagging with an R^2^ above 0.8. Genotyping was performed using Taqman allelic discrimination (KBioscience, Hoddesdon, UK) with a genotyping success rate above 97% for all five variants and only one genotyping error was observed out of a total of 1,726 duplicate samples. All genotype groups obeyed Hardy-Weinberg equilibrium in the population-based sample of Danes (*p*>0.2).

### Statistical analysis

LD-structure was generated using HaploView 4.1. Fisher's exact test was applied to analyse differences in minor allele frequency as well as genotype distribution of the individual genotypes. Logistic regression assuming an additive model was performed to evaluate the difference in genotype distribution adjusted for sex and age in relation to obesity and hypertensive status. Multiple linear regression analysis was used to analyse the effect of individual genotypes on quantitative traits. Haplotype frequencies were estimated using the expectation-maximization (EM) algorithm and association was evaluated by global and haplotype-specific score statistics [10]. The global *p*-value is an estimate of the overall effect of haplotypes in the statistical model and the specific *p*-value is an estimate of the effect of a specific haplotype compared to the effect of the remaining haplotypes. Haplotypes with a frequency below 0.5% were excluded. The program PHASE version 2.1.1 was used to estimate the combinations of haplotypes. PHASE is a program using a Bayesian statistical method for reconstructing haplotypes from population genotype data developed by Stephens and colleagues [11], [12]. General linear models with adjustment for sex and age was used to test a reference haplotype combination against the remaining haplotype combinations (in a pair-wise manner) for differences in the frequency of obesity and hypertension and traits related to these conditions and a specific *p*-value for each comparison was calculated. The reference was set as the haplotype combination (4,4). This was chosen as the reference based on the decreased expression previously seen for the haplotype corresponding to haplotype number 4 in the present study [4]. F-test (for continuous traits) and Chi-squared (for binary traits) were used to calculate the global *p*-value representing the fit to the model added by haplotype combinations compared to a model including only sex, age and BMI. Statistical analyses rely on traits being normally distributed which after evaluation of quantile-quantile plots were found. Patients with known diabetes (*n* = 108) were excluded from all quantitative trait analyses and patients receiving anti-hypertensive treatment (*n* = 403) were excluded from quantitative trait analyses of blood pressure. A statistical power calculation was performed assuming haplotype frequencies of 39% versus 27% as observed in a previous report [6], a significance level of 95%, and a sample size of 1,000. All analyses were performed using RGui version 2.7. A *p*-value of less than 0.05 was considered to be significant.

## Results

The five *ADRB2* variants which were genotyped were all located within the coding region and their allele frequencies were: 38% for rs1042713; 45% for rs1042714; 1.5% for rs1800888; 15% for rs1042718 and 28% for rs1042719. The LD-structure for the SNPs was evaluated and D' was above 0.8 except for the two variations located furthest apart (Figure S1). R^2^ was below 0.8 for all analyses (Figure S1). Only variants located within the same LD-block were included in the haplotypes. Thus, haplotypes were constructed from rs1042713, rs1042714, rs1800888, and rs1042718. These haplotypes were examined for association with obesity (Table 1) however; both specific and global effects were insignificant. Nor was any association of the haplotypes observed with BMI, waist circumference, or body weight (data not shown).

**Table 1:** *ADRB2* haplotype analysis comparing obesity and hypertension status among 5,838 and 5,584 individuals from the Inter99 study sample, respectively.

Number corresponding to (2).	rs1042713 (Gly16Arg)	rs1042714 (Gln27Glu)	rs1800888 (Thr164Ile)	rs1042718 (AA175 Synonymous)	Frequency (%)	Obesity Specific *p*-value	Hypertension Specific *p*-value
2	G	G	C	C	44	0.5	0.6
5	G	C	C	C	3	0.7	0.9
7	G	C	T	A	2	0.8	1.0
6	G	C	C	A	13	0.9	0.1
4	A	C	C	C	38	0.5	0.1
**Global ** ***p*** **-value**						1.0	0.4

Haplotype frequencies were estimated using the expectation-maximization (EM) algorithm and association was evaluated by global and haplotype-specific score statistics (Schaid et al.2002). The global *p*-value is an estimate for the overall effect of haplotypes in the statistical model and the specific *p*-value is an estimate of the effect of a specific haplotype compared to the effect of the remaining haplotypes combined. Obesity status was examined as individuals having a BMI below 25 kg/m^2^ vs. individuals having a BMI above 30 kg/m^2^. Hypertension status was examined as mean systolic blood pressure≥140 mm Hg and/or mean diastolic blood pressure≥90 mm Hg and/or current or previous treatment with antihypertensive drugs vs. normotensive individuals.

**Figure 1:**
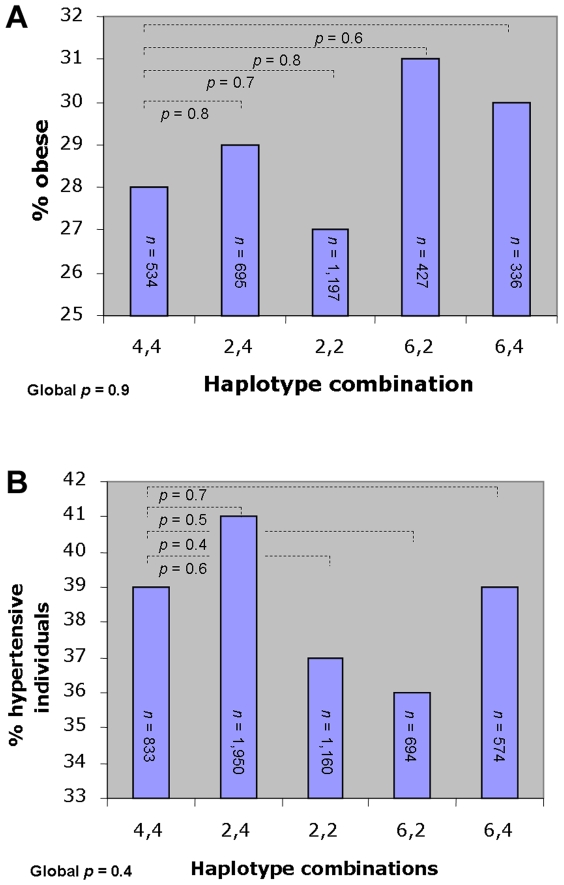
Case-control study comparing the frequency of obese and hypertensive individuals among the *ADRB2* haplotype pairs, respectively. A: The proportion of obese out of the combined number of individuals in the lean group (BMI<25 kg/m^2^) and the obese (BMI>30 kg/m^2^) group (*n* = 3,189). B: The proportion of hypertensive individuals out of the combined number of individuals in the normotensive group and the hypertensive group (*n* = 5,211).

Due to the diploid nature of the human genome the haplotype combination of each participant was assessed. A total of 21 haplotype combinations were present. Five combinations had a frequency above 5% and these were selected for further analysis. These combinations were examined in a case-control study comparing pair-wise the frequencies of obese versus lean individuals for each combination against a reference combination (Figure 1A). This analysis did not detect any effect of the haplotype pairs, a finding which was supported by a global analysis (*p* = 0.9). When pair-wise comparisons of BMI, waist circumference and waist/hip ratio were performed, these combined haplotypes only revealed a nominal difference in waist-to-hip ratio between the reference and the haplotype combination 6,2, which had increased waist-to-hip ratio (*p* = 0.02; Table 2).

**Table 2:** Comparison between *ADRB2* haplotype combinations and body composition and blood pressure among 4,885 and 4,566 Danish individuals, respectively.

Haplotype combination	4,4	2,4	2,2	6,2	6,4
**BMI (kg/m2)**	26.1±4.8	26.3±4.7	26.2±4.3	26.0±4.4	26.2±4.9
Specific *p*-value	-	0.3	0.6	0.9	0.9
Global *p*-value	0.8				
**Waist (cm)**	86.0±13.4	86.6±13.3	86.3±12.9	86.5±13.3	86.4±13.2
Specific *p*-value	-	0.3	0.3	0.5	0.7
Global *p*-value	0.8				
**Waist/hip ratio**	0.85±0.09	0.85±0.08	0.85±0.09	0.86±0.09	0.86±0.09
Specific *p*-value	-	0.7	0.4	**0.02**	0.7
Estimate of effect				0.008	
Global *p*-value	0.1				
**Systolic blood pressure (mm Hg)**	128.1±16.0	129.7±17.0	128.9±16.5	129.6±16.8	129.0±17.3
Specific *p*-value	-	**0.04**	0.2	0.1	0.5
Estimate of effect		1.40			
Global *p*-value	0.3				
**Diastolic blood pressure (mm Hg)**	81.2±10.7	82.1±11.1	81.6±10.9	82.0±11.0	81.5±11.3
Specific *p*-value	-	0.09	0.3	0.2	0.7
Estimate of effect					
Global *p*-value	0.4				
**MABP**	96.8±11.6	98.0±12.3	97.4±11.9	97.9±12.2	97.4±12.6
Specific *p*-value	-	**0.05**	0.2	0.1	0.6
Estimate of effect		0.99			
Global *p*-value	0.3				

Data is mean±SD. P-values were corrected for age and sex. MABP (mean arterial blood pressure) is calculated as [(2×diastolic blood pressure)+systolic blood pressure] divided by 3. The specific *p*-value is calculated based on a pair-wise comparison of the haplotype combination with the reference haplotype combination (4,4) using a multiple linear regression and the global *p*-value has been calculated using an ANOVA. Patients with known diabetes were excluded from all of the above analyses and patients receiving anti-hypertensive treatment were excluded from blood pressure trait analyses.

The haplotypes and haplotype combinations were also examined for association with hypertension. Haplotype analyses yielded the same lack of association with hypertension and blood pressure (Table 1). Also, haplotype combinations failed to significantly associate with hypertension (Figure 1B). However, a nominal significant difference in measures of systolic blood pressure as well as mean arterial blood pressure was observed between the reference combination and haplotype combination 2,4 with the latter having an increased blood pressure (Table 2).

Interactions between sex and the individual variants as well as haplotype pairs were examined (data not shown). Minor interaction was seen between rs1042719 and sex for measures of blood pressure; however, when performing sex stratified analyses for this variant no significant associations was found (data not shown). In contrast, we did not observe any interaction between sex and the effect of the haplotype pairs.

All of the above analyses were also performed after removal of the rare rs1800888, yet no robust associations were found except for an association with waist-to-hip ratio of a haplotype pair corresponding to 6,2 excluding rs1800888 (data not shown).

Despite the overall aim to evaluate the effect of haplotype combinations, the effect of individual genotypes was examined, yet no effect on either obesity or hypertension was observed for rs1800888, rs1042718, and rs1042719 (Table S1). The same lack of association was observed for the quantitative traits study (Table S2), except for a nominal association with increased waist-to-hip ratio for rs1042718 (*p* = 0.03).

## Discussion

No consistent association with body weight or blood pressure regulation was found for the individual *ADRB2* locus haplotypes. Neither did analyses of *ADRB2* haplotype combinations indicate any physiological impact on weight status except from the nominal difference in waist-to-hip ratio between the reference and the haplotype combination 6,2.

If the haplotype previously showing a decreased expression (haplotype number 4) had a physiologically impact on weight regulation, we would expect this haplotype to be associated with increased BMI, waist circumference or waist-hip ratio or an increased proportion of obese individuals due to a reduced lipolysis. Or it could have been expected that the haplotype combination showing the lowest response to a ADRB2 agonist (4,4) lead to a higher BMI due to a reduced agonist response and thus lower lipolysis. Yet, this is not the case.

Examination of the influence of haplotype combinations on measures of blood pressure showed a nominal effect of the haplotype combination 2,4 compared to haplotype pair 4,4. This combination is composed of the haplotypes previously showing the highest (2) and the lowest expression (4) of mRNA and protein *in vitro*, thus there is no apparent link between expression and the physiological response observed. Also, no clear relation was seen between the haplotype combination (2,4) associating with increased blood pressure in the present study - most likely caused by vessel contraction - and the haplotype combination (6,6) previous associating with increased noradrenalin sensitivity measured in fat cells as maximum effect on lipolysis [5].

A statistical power calculation was performed based on the previously reported effect of *ADRB2* haplotypes on obesity (OR: 0.58; CI: 0.34–0.96) [6]. In our study we have a statistical power of 1 to detect an equivalent association. If the haplotype pairs showing nominal effects on waist-to-hip ratio (4,4 and 6,2) and systolic blood pressure (4,4 and 2,4) are used for obesity and hypertension case-control studies, we can based on the OR confidence intervals estimate that it is unlikely in the present study population to find effect sizes on obesity above 1.4 and above 1.3 on hypertension.

Marker-by-marker analyses of rs1800888, rs1042718 and rs1042719 of *ADRB2* in the present study and the previous published analyses of rs1042713 and rs1042719 [4] showed nominally significant association with waist-to-hip ratio and systolic blood pressure in line with the results seen for haplotype pairs.

In summary, based on the findings of the present population based study in 6,514 adult Danes there is no evidence of functional haplotypes in the *ADRB2* locus with significant impact on body fatness or blood pressure regulation.

## Supporting Information

Table S1 Case-control studies examining the individual effects of variants in *ADRB2* on obesity and hypertension among 6,514 individuals from the Inter99 study sample.
(0.08 MB DOC)

Table S2 Quantitative trait analyses associations between 3 common *ADRB2* variants and measures related to obesity and hypertension among 6,039 and 5,638 Danes from the inter99 study sample, respectively.
(0.08 MB DOC)

Figure S1 Pair-wise LD plot calculated for the present study population of 5,730 Danes for the five *ADRB2* variants estimated using D' (A) and R^2^ (B).
(7.65 MB TIF)
