# Supplementary material for: No Consistent Effect of ADRB2 Haplotypes on Obesity, Hypertension and Quantitative Traits of Body Fatness and Blood Pressure among 6,514 Adult Danes
Source: PLoS One. 2009 Sep 25;4(9):e7206. doi: 10.1371/journal.pone.0007206 (PMC2745753; doi:10.1371/journal.pone.0007206)
Supplement: Table S1 — Case-control studies examining the individual effects of variants in ADRB2 on obesity and hypertension among 6,514 individuals from the Inter99 study sample. (0.08 MB DOC) [file pone.0007206.s001.doc]

|  | **BMI < 25 kg/m^2^** | **BMI > 30 kg/m^2^** | ***P*_Fishers_** | **OR_Fishers_** | ***P*_GLM_** | **OR_GLM_** | **Normotensive** | **Hypertensive** | ***P*_Fishers_** | **OR_Fishers_** | ***P*_GLM_** | **OR_GLM_** |
| --- | --- | --- | --- | --- | --- | --- | --- | --- | --- | --- | --- | --- |
| **1042718** |  |  |  |  |  |  |  |  |  |  |  |  |
| **CC (%)** | 1977 (73.2) | 767 (72.3) |  |  |  |  | 2704 (71.9) | 1765 (73.8) |  |  |  |  |
| **CA (%)** | 668 (24.7) | 271 (25.5) |  |  |  |  | 984 (26.1) | 574 (24) |  |  |  |  |
| **AA (%)** | 55 (2) | 23 (2.2) |  |  |  |  | 75 (2) | 54 (2.3) |  |  |  |  |
| **MAF**  **(95% CI)** | 14.4 (13.5-15.3) | 14.9 (13.4-16.5) | 0.6 | 1.04 (0.9-1.2) |  |  | 15.1 (14.3-15.9) | 14.1 (13.1-15.1) | 0.2 | 0.94 (0.84-1.04) |  |  |
| **Genotype distribution** |  |  | 0.8 |  | 0.8 | 1.02 (0.88-1.18) |  |  | 0.1 |  | 0.1 | 0.92 (0.82-1.02) |
| **Rs1042719** |  |  |  |  |  |  |  |  |  |  |  |  |
| **CC (%)** | 1386 (51.4) | 544 (51.4) |  |  |  |  | 1944 (51.8) | 1243 (52) |  |  |  |  |
| **CA (%)** | 1096 (40.7) | 428 (40.5) |  |  |  |  | 1512 (40.3) | 956 (40) |  |  |  |  |
| **AA (%)** | 212 (7.9) | 86 (8.1) |  |  |  |  | 296 (7.9) | 191 (8) |  |  |  |  |
| **MAF (95% CI)** | 28.2 (27.0-29.4) | 28.4 (26.4-30.3) | 0.9 | 1.01 (0.9-1.13) |  |  | 28.0 (27.0-29.1) | 28.0 (26.7-29.3) | 1.0 | 1.0 (0.92-1.08) |  |  |
| **Genotype distribution** |  |  | 1.0 |  | 0.8 | 1.01 (0.9-1.14) |  |  | 1.0 |  | 0.9 | 1.0 (0.92-1.09) |
| **Rs1800888** |  |  |  |  |  |  |  |  |  |  |  |  |
| **CC (%)** | 2611 (96.8) | 1022 (96.9) |  |  |  |  | 3642 (96.9) | 2332 (97.2) |  |  |  |  |
| **CA (%)** | 85 (3.2) | 33 (3.1) |  |  |  |  | 115 (3.1) | 66 (2.8) |  |  |  |  |
| **AA (%)** | 1 (0) | 0 (0) |  |  |  |  | 1 (0) | 1 (0) |  |  |  |  |
| **MAF (95% CI)** | 1.6 (1.3-1.9) | 1.6 (1.0-2.1) | 0.9 | 0.97 (0.63-1.47) |  |  | 1.6 (1.3-1.8) | 1.4 (1.1-1.8) | 0.6 | 0.9 (0.7-1.24) |  |  |
| **Genotype distribution** |  |  | 1.0 |  | 0.8* | 1.04 (0.68-1.58) |  |  | 0.8 |  | 1.0* | 1.0 (0.72-1.39)* |

Data are number of subjects in each genotype group (% of each group) and minor allele frequency (MAF) in % (95% CI). The *p*-values were calculated using Fisher’s exact test (Fishers) for the genotype distribution and the allele frequency and using a general linear model (GLM) for genotype distribution correcting for age and sex.* Dominant model due to the low number of homozygous individuals.
