# Supplementary material for: No Consistent Effect of ADRB2 Haplotypes on Obesity, Hypertension and Quantitative Traits of Body Fatness and Blood Pressure among 6,514 Adult Danes
Source: PLoS One. 2009 Sep 25;4(9):e7206. doi: 10.1371/journal.pone.0007206 (PMC2745753; doi:10.1371/journal.pone.0007206)
Supplement: Table S2 — Quantitative trait analyses associations between 3 common ADRB2 variants and measures related to obesity and hypertension among 6,039 and 5,638 Danes from the inter99 study sample, respectively. (0.08 MB DOC) [file pone.0007206.s002.doc]

| **Rs 1800888** | **CC** | **CT** | **TT** | ***P*** |
| --- | --- | --- | --- | --- |
| ***n*** | 5605 | 170 | 2 |  |
| **Age (years)** | 46 ± 8 | 45 ± 8 | 40 ± 7 |  |
| **BMI (kg/m^2^)** | 26.2 ± 4.5 | 26.1 ± 4.7 | 23.8 ± 5.5 | 1.0* |
| **Waist (cm)** | 86 ± 13 | 86 ± 14 | 82 ± 23 | 0.7* |
| **Waist/hip ratio** | 0.86 ± 0.09 | 0.85 ± 0.08 | 0.82 ± 0.18 | 0.7* |
| **Systolic BP (mm Hg)** | 129 ± 17 | 127 ± 16 | 132 ± 18 | 0.6* |
| **Diastolic BP (mm Hg)** | 82 ± 11 | 82 ± 11 | 78 ± 14 | 0.6* |
| **Rs 1042718** | **CC** | **CA** | **AA** | ***P*** |
| *n* | 4196 | 1454 | 120 |  |
| **Age (years)** | 46 ± 8 | 46 ± 8 | 46 ± 8 |  |
| **BMI (kg/m^2^)** | 26.2 ± 4.6 | 26.1 ± 4.4 | 26.2 ± 4.4 | 0.4 |
| **Waist (cm)** | 86 ± 13 | 86 ± 13 | 87 ± 13 | 0.4 |
| **Waist/hip ratio** | 0.85 ± 0.09 | 0.86 ± 0.09 | 0.86 ± 0.09 | **0.03** |
| **Systolic BP** | 129 ± 17 | 129 ± 17 | 127 ± 17 | 0.2 |
| **Diastolic BP** | 82 ± 11 | 82 ± 11 | 81 ± 12 | 0.3 |
| **Rs 1042719** | **GG** | **GC** | **CC** | ***P*** |
| **n** | 3003 | 2298 | 459 |  |
| **Age (years)** | 46 ± 8 | 46 ± 8 | 46 ± 8 |  |
| **BMI (kg/m^2^)** | 26.3 ± 4.5 | 26.2 ± 4.6 | 26.1 ± 4.4 | 0.4 |
| **Waist (cm)** | 87 ± 13 | 86 ± 13 | 86 ± 13 | 0.8 |
| **Waist/hip ratio** | 0.86 ± 0.09 | 0.86 ± 0.09 | 0.86 ± 0.09 | 0.8 |
| **Systolic BP** | 129 ± 17 | 129 ± 17 | 128 ± 16 | 0.5 |
| **Diastolic BP** | 82 ± 11 | 82 ± 11 | 82 ± 11 | 0.7 |

Data is mean ± SD. *P*-values were corrected for age and sex. Patients with known diabetes were excluded from all of the above analyses and patients receiving anti-hypertensive treatment were excluded from blood pressure quantitative trait analyses. *Analyses have been conducted using a dominant model
