# Supplementary figures and images for: No Consistent Effect of ADRB2 Haplotypes on Obesity, Hypertension and Quantitative Traits of Body Fatness and Blood Pressure among 6,514 Adult Danes
Source: PLoS One. 2009 Sep 25;4(9):e7206. doi: 10.1371/journal.pone.0007206 (PMC2745753; doi:10.1371/journal.pone.0007206)

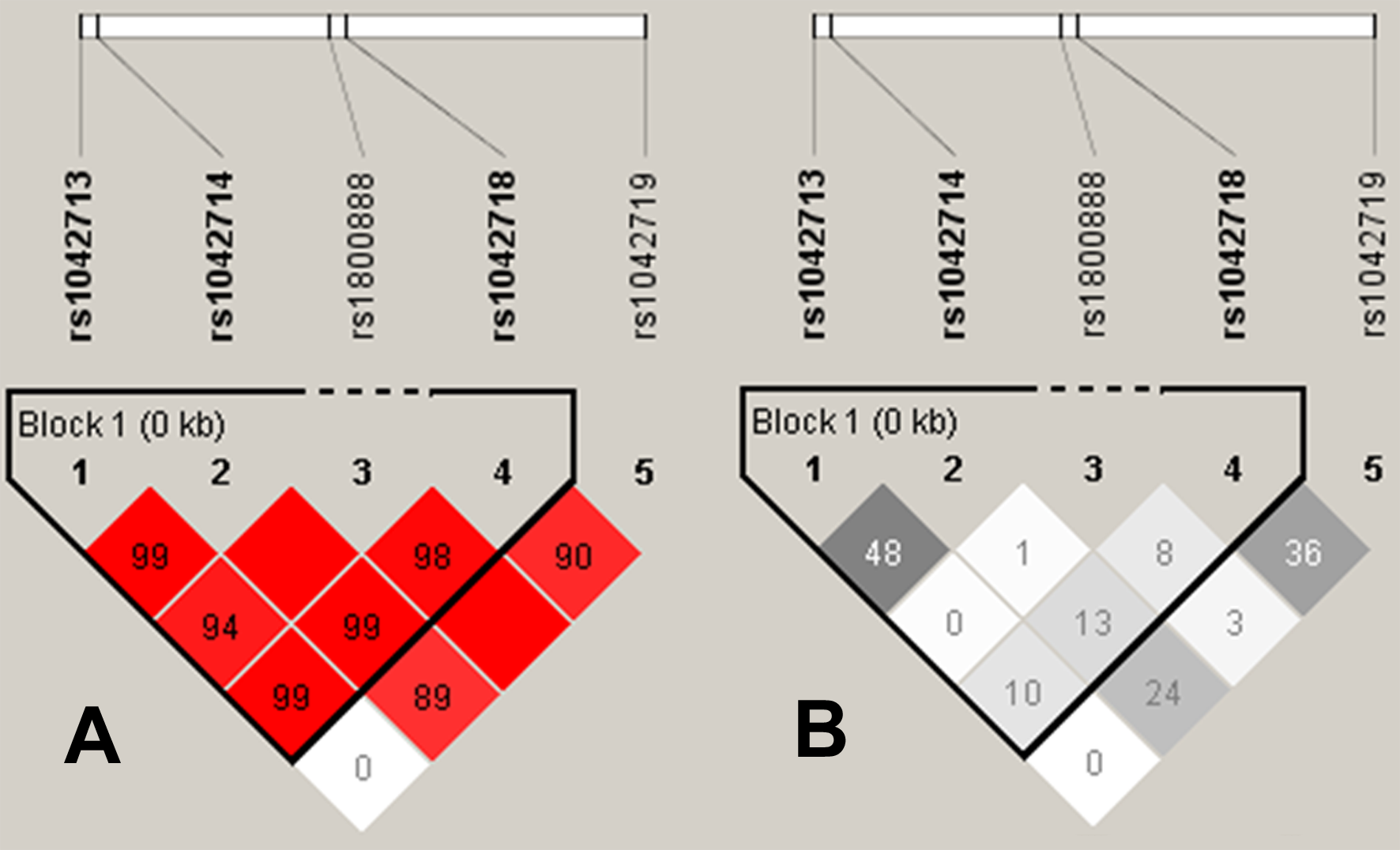

Supplement: Figure S1 — Pair-wise LD plot calculated for the present study population of 5,730 Danes for the five ADRB2 variants estimated using D' (A) and R2 (B). (7.65 MB TIF) [file pone.0007206.s003.tif]
